# Disseminated cutaneous mast cell tumor in a South American coati (*Nasua nasua*)

**DOI:** 10.1007/s11259-026-11443-6

**Published:** 2026-07-31

**Authors:** Renata Dalcol Mazaro, Gabriela Hartmann, Diego Cardoso, Tereza Cristina Da Silva, Bruno Cogliati, Glaucia Denise Kommers, Rafael Almeida Fighera

**Affiliations:** 1 Laboratório de Patologia Veterinária, Universidade Federal de Santa Maria, Avenida Roraima 1000 – Camobi, Santa Maria , RS 97105-900 Brazil; 2 Setor de Patologia Veterinária, Faculdade de Veterinária, Universidade Federal do Rio Grande do Sul, Avenida Bento Gonçalves 9090 – Agronomia, Porto Alegre, RS 90650-001 Brazil; 3 Jardim Botânico e Zoológico Municipal de Cachoeira do Sul, Silvio Scopel 502 – Centro, Cachoeira do Sul, RS 96506-630 Brazil; 4 Laboratório de Patologia Morfológica e Molecular, Faculdade de Medicina Veterinária e Zootecnia, Universidade de São Paulo, Avenida Prof. Dr. Orlando Marques de Paiva 87 – Butantã, São Paulo, 05508-270 SP Brazil

**Keywords:** Round cell tumors, Cancer, Skin, Procyonids

## Abstract

Cutaneous mast cell tumors have been described in all species of domestic mammals. However, information about these tumors in free-ranging and captive wild mammals is limited, with no reports in procyonids. This report aims to describe a case of disseminated cutaneous mast cell tumor in a captive South American coati (*Nasua nasua*). The animal was submitted to euthanasia and necropsy. On external examination, multiple papules, nodules, and plaques were distributed over the body surface. On cut section, they were soft and white. Histologically, they were composed of dense sheets of neoplastic cells interspersed with a delicate collagenous stroma. Cells were round and had a finely granular cytoplasm. Mast cell tumors were diagnosed based on histological features, positive staining with toluidine blue, and immunohistochemical labelling for the KIT protein. To the best of our knowledge, this is the first report of disseminated cutaneous mast cell tumor in this animal species.

## Background

Cutaneous mast cell tumors have been described in all species of domestic mammals but are more common in dogs, cats, and ferrets (Avallone et al. 2016; Kiupel 2017). The clinical presentation varies from single to multiple lesions appearing as small papules or nodules (that can be alopecic, erythematous, or hyperpigmented) to large plaques or masses, covered by intact or ulcerated skin (Avallone et al. 2016; Gross et al. 2005; Kiupel 2017). Biological behavior varies among animal species and tumor locations. Prognostic factors are currently well established for canine mast cell tumors, based on histological grade and molecular characteristics (Kiupel 2017). However, information about these tumors free-ranging and captive wild mammals is limited with no reports in procyonids. This report aims to describe a case of disseminated cutaneous mast cell tumor in a South American coati (*Nasua nasua*).

## Case presentation

**Fig. 1:**
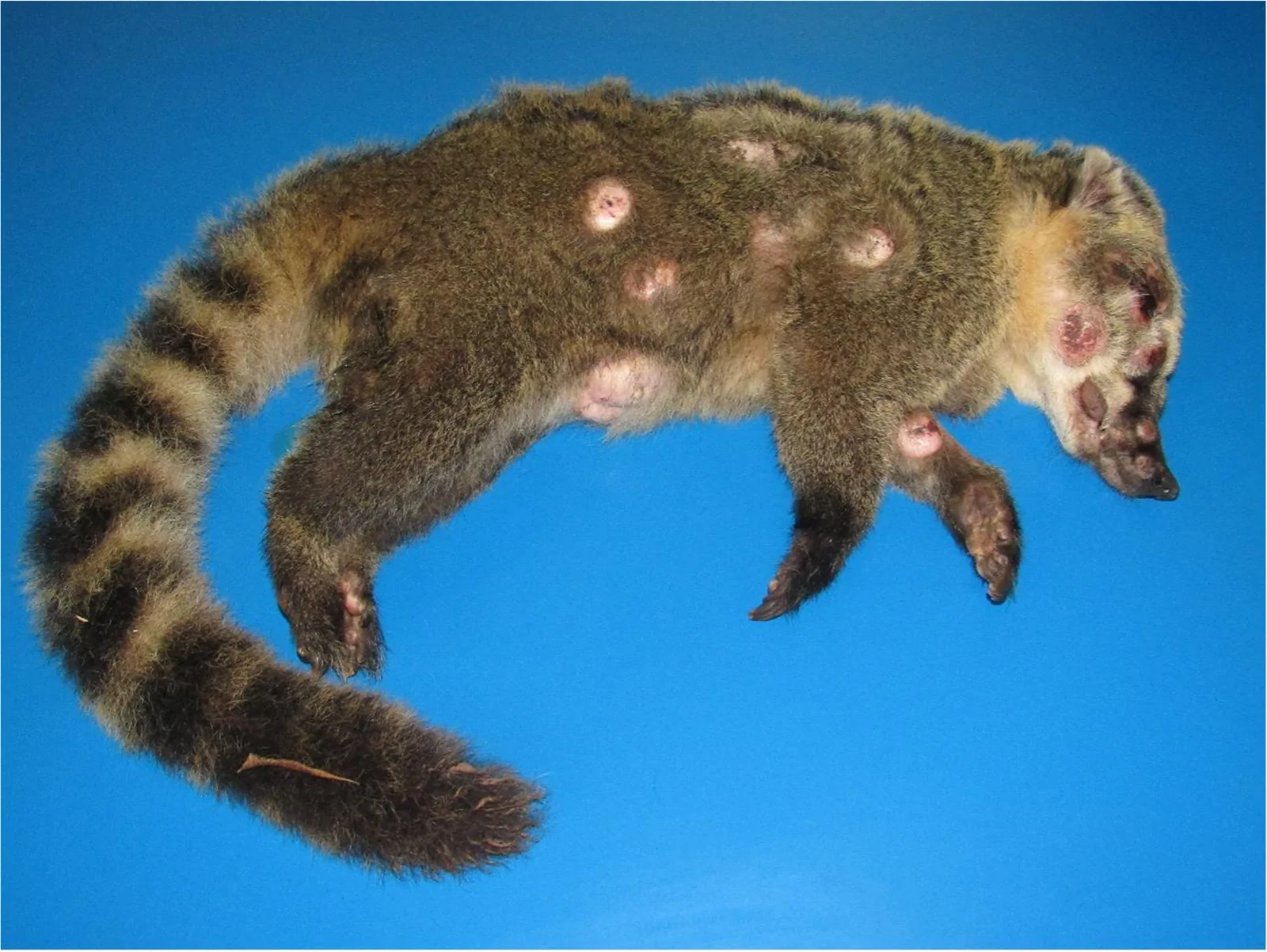
Disseminated cutaneous mast cell tumors in an old female South American coati (*Nasua nasua*). Multiple alopecic, pink, and partially ulcerated cutaneous nodules were distributed over the body surface, primarily in the head and trunk

**Fig. 2:**
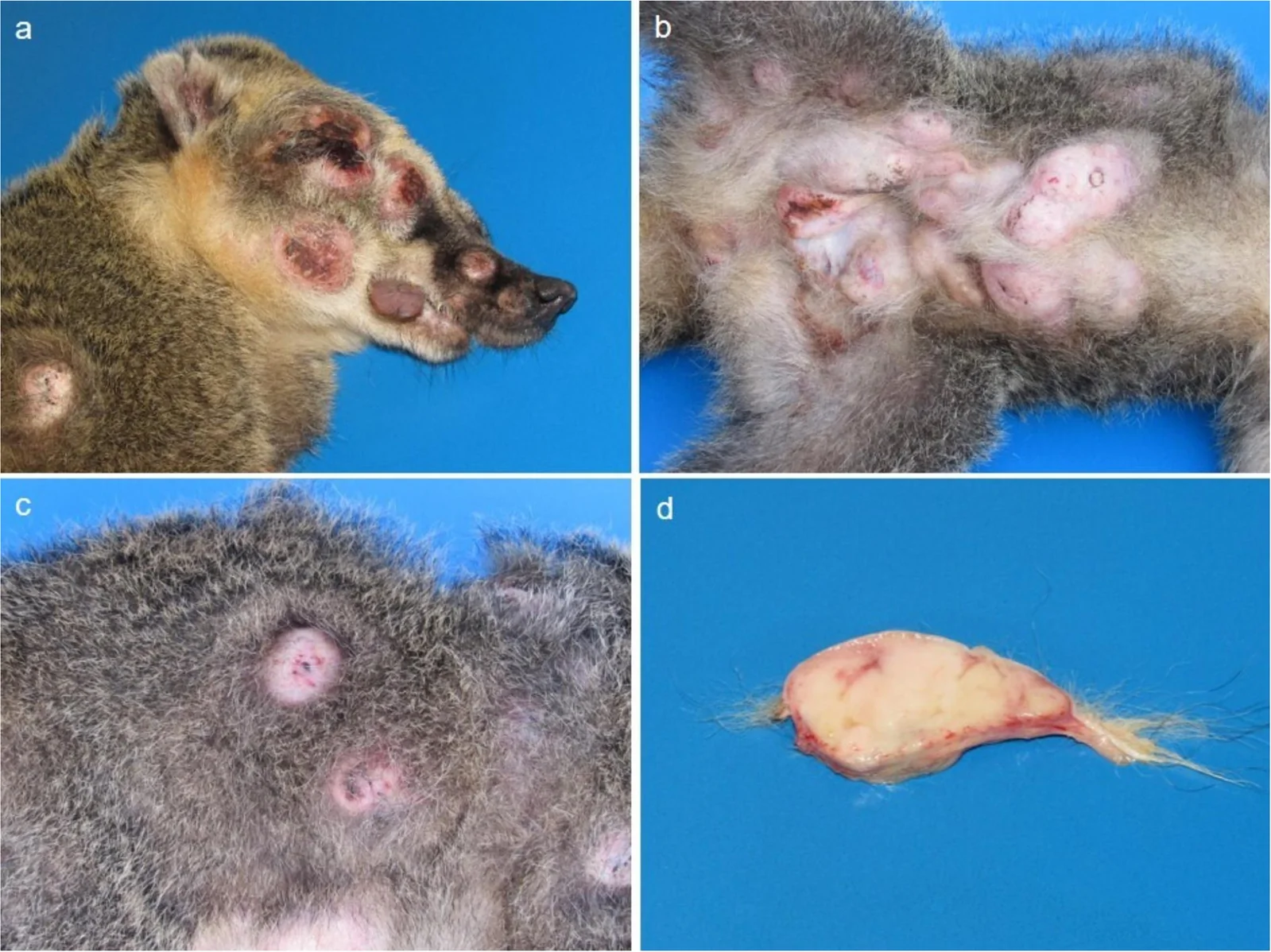
Disseminated cutaneous mast cell tumors in an old female South American coati (*Nasua nasua*). (**a**) Face showing multiple nodules and plaques, most of them are ulcerated and covered with hemorrhagic crusts. (**b**) Coalescing plaques on the chest, ventral abdomen, and inguinal region in the mammary gland area. (**c**) Close-up view of a homogeneously pink and centrally ulcerated nodule on the right flank. (**d**) The white cut surface of a skin plaque

A 15-year-old female captive South American coati (*Nasua nasua*) presented with an erythematous papule on its left upper eyelid. The lesion progressed to generalized skin involvement in about 6 months. No treatment was performed. Due to the poor clinical condition, the animal was euthanized and submitted to necropsy. On external examination, multiple papules, nodules, and plaques were distributed over the body surface, predominantly on the trunk (Fig. 1) and face (Figs. 1 and 2a). Additionally, some nodules and plaques coalesced to form large masses in the ventral thoracic region (Fig. 2b). The lesions were alopecic, pink, occasionally ulcerated. They ranged from 0.5 cm in diameter to 7 × 5.5 × 2 cm (Fig. 2a and b, and 2c). On cut section, they were soft and white (Fig. 2d). Larger lesions invaded subcutaneous tissue and, sometimes, the adjacent muscles. Other organs were grossly unremarkable. Samples of tumors and multiple organs, including brain, heart, tonsils, lungs, liver, spleen, stomach, pancreas, intestines, kidneys, adrenals, urinary bladder, lymph nodes, bone marrow, and thyroid were fixed in 10% buffered formalin, processed routinely, embedded in paraffin wax, cut at 3 μm and stained with hematoxylin and eosin.

**Fig. 3:**
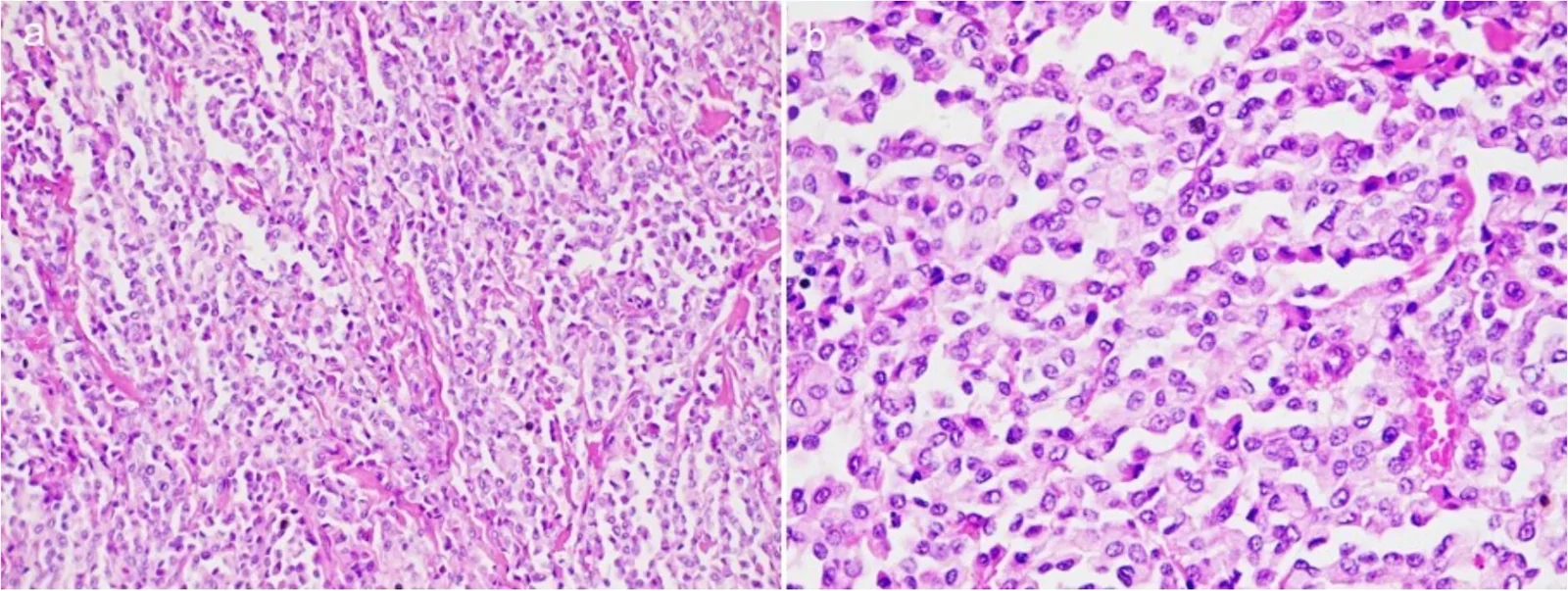
Mast cell tumor in an old female South American coati (*Nasua nasua*). Skin. (**a**) Dense round cell population arranged in sheets, interspersed with thin collagenous stroma. Hematoxylin and Eosin. (**b**) High magnification of Fig. 3a. Round neoplastic cells with finely granular cytoplasm and mostly eccentric nuclei containing loose chromatin. Hematoxylin and Eosin

Histologically, each cutaneous lesion was rather well demarcated, non-encapsulated and composed of a densely cellular neoplastic population extending from the superficial to the deep dermis. In some sections, the subcutaneous tissue and adjacent muscles were also affected. Neoplastic cells were arranged in sheets, interspersed with a delicate collagenous stroma (Fig. 3a). The neoplastic cells were round with a finely granular cytoplasm. The nuclei were round or oval, with most of them being slightly eccentric and having vesicular nuclei. The nucleoli were single, small, basophilic, and variably evident (Fig. 3b). Binucleated neoplastic cells were common, while multinucleated cells were occasionally observed. There was mild pleomorphism, moderate atypia, and two mitotic figures (in a 2.37 mm^2^ area). Rare eosinophils were observed intermingled with neoplastic cells. All other organs collected were examined histologically, but no metastasis was observed.

**Fig. 4:**
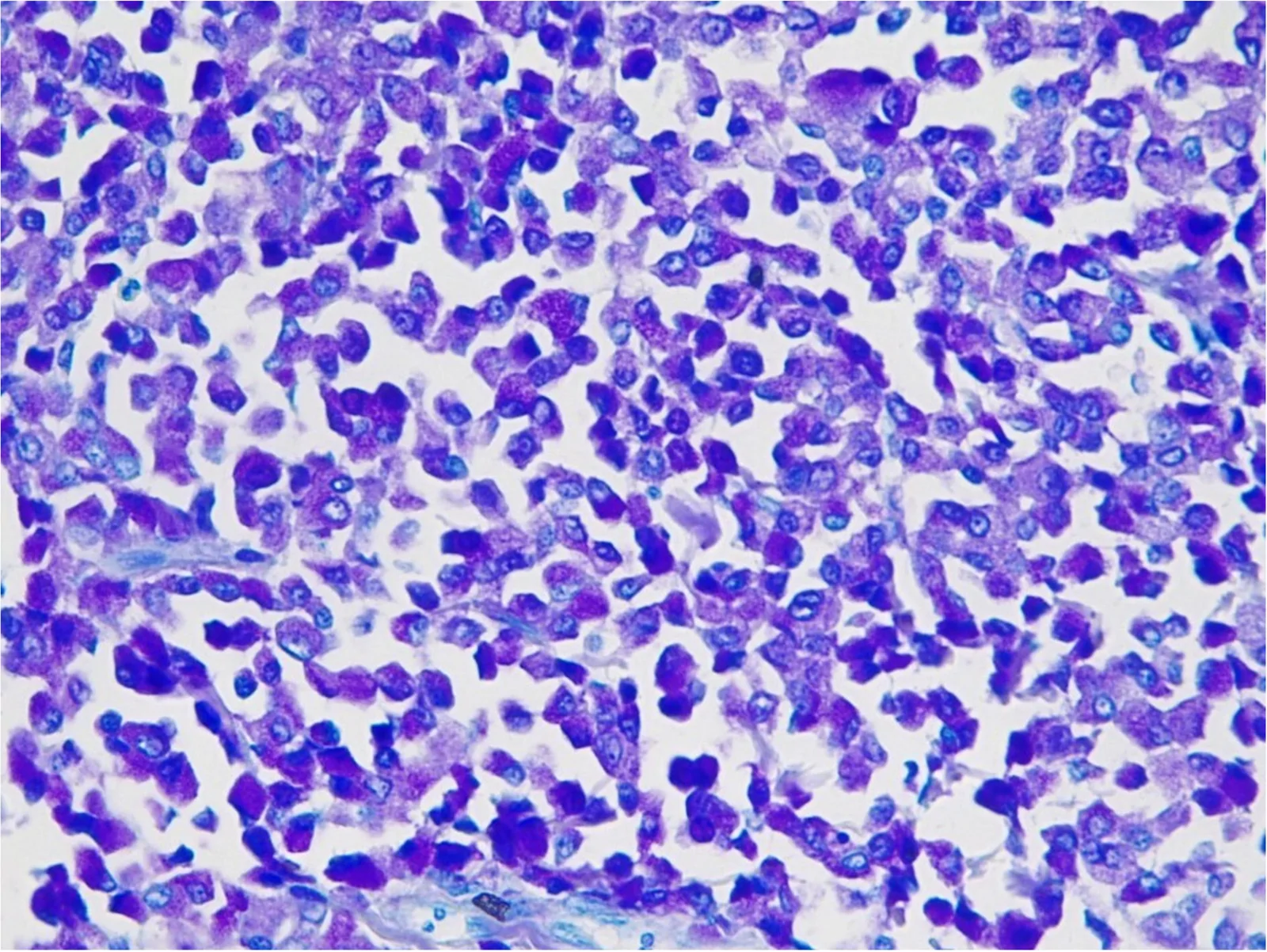
Mast cell tumor in an old female South American coati (*Nasua nasua*). Skin. Dense cytoplasmic metachromatic granules in the neoplastic mast cells. Toluidine blue staining

**Fig. 5:**
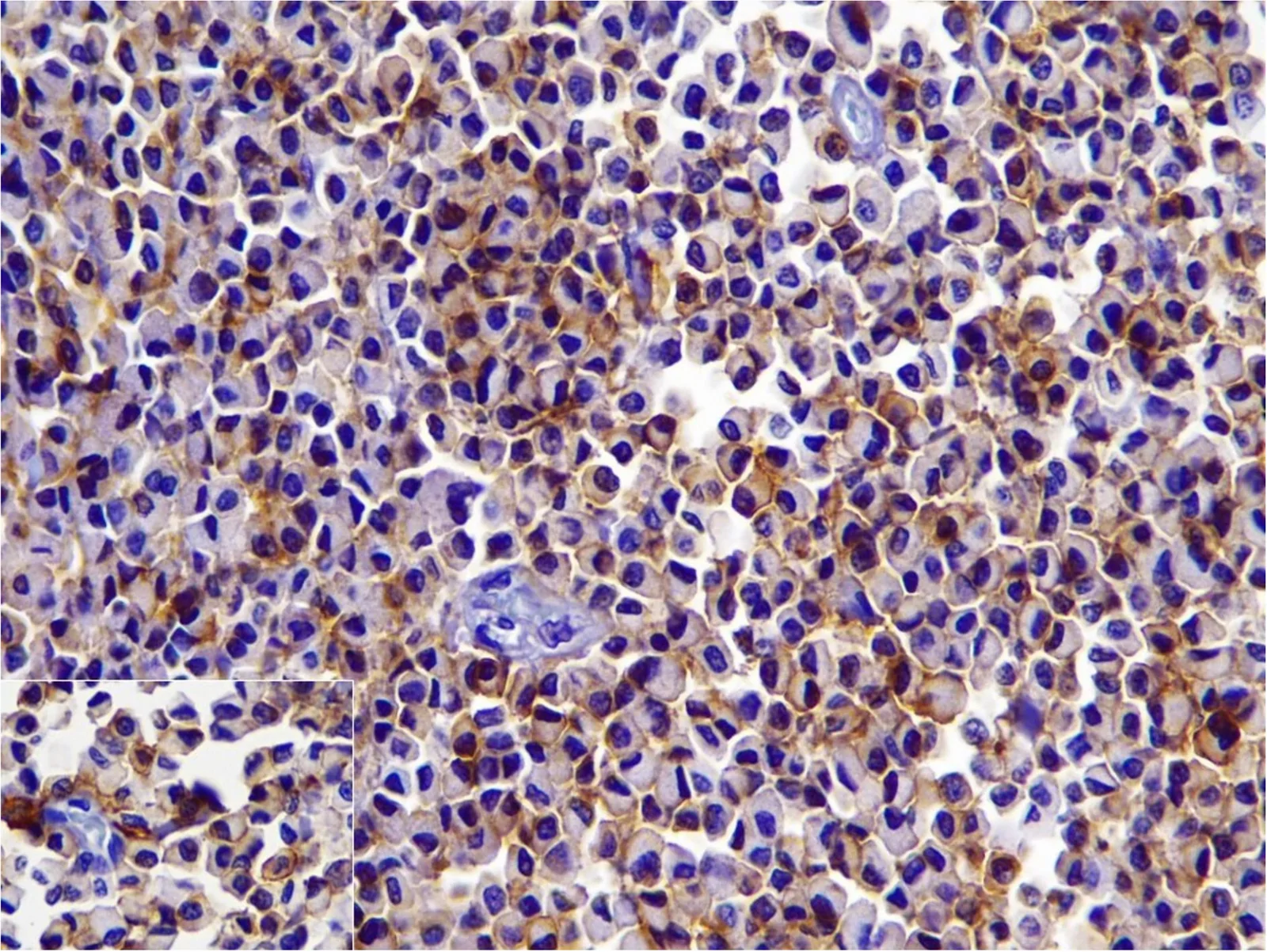
Mast cell tumor in an old female South American coati (*Nasua nasua*). Skin. The KIT-positive immunostained neoplastic mast cells with a membrane-associated pattern and minor cytoplasmic staining. Inset, picture with higher magnification. Immunohistochemistry technique

Based on these findings, a mast cell neoplasm was suspected. Toluidine blue stain confirmed the presence of cytoplasmic metachromatic granules in the neoplastic cells (Fig. 4). Immunohistochemistry with anti-CD117 antibody was used to assess the expression of the KIT protein. Neoplastic cells showed a membrane-associated immunostaining pattern with minimal cytoplasmic staining (Fig. 5). The findings led to a final diagnosis of disseminated cutaneous mast cell tumor. The immunohistochemical technique employed was performed according to the following protocol: After tissue deparaffinization and rehydration, antigen recovery using Tris-EDTA solution (pH 9.0) was performed in pressure cooker oven for 15 min. Endogenous peroxidase blockade was performed using 3% hydrogen peroxide for 20 min. Nonspecific reactions were blocked using Protein Block at room temperature for 10 min. The primary antibody was anti-CD117 polyclonal (1:200 dilution, Dako A-4502, produced in rabbit, anti-human). The antibody was incubated in refrigerator at 7 °C for 16 h (overnight). Secondary antibody and Horseradish peroxidase (HRP) polymer were used consecutively incubated at 25 °C for 30 min and developed by adding 3–3’diaminobenzidine (DAB) tetrachloride chromogen for 5 min. Counterstaining was performed using Harris Hematoxylin. A canine cutaneous mast cell tumor was used as positive and negative controls. Negative control was obtained by omitting the primary antibody using only the antibody diluent.

## Discussion and conclusions

Scarce descriptions of cutaneous mast cell tumors in wild mammals and unconventional pets include a kowari (*Dasyuroides byrnei*) (Madsen et al. 2017), winter white dwarf hamster (*Phodopus sungorus*) (Nishizumi et al. 2000), four-toed hedgehog (*Atelerix albiventris*) (Madsen et al. 2017), Richardson’s ground squirrel (*Urocitellus richardsonii*) (He et al. 2009), African lion (*Panthera leo*) (Stolte and Welle 1995), tiger (*Panthera tigris*) (Kloft et al. 2019), cheetah (A*cinonyx jubatus*) (Campbell-Ward et al. 2021), and llama (*Lama glama*) (Lin et al. 2010). Members of the Procyonidae family can develop similar neoplasms that affect domestic carnivores (Church et al. 2018); however, epitheliotropic cutaneous lymphoma (mycosis fungoides) is the only round cell cutaneous tumor described in a coati to date (Skorinsky et al. 2008). Based on the fact that no previous descriptions were found in the veterinary literature, the authors believe this to be the first case of cutaneous mast cell tumor in South American coati.

Based on the pattern of lesion distribution on the skin (multiple coalescing nodules) and on histopathological findings, the differential diagnoses considered for this case include mast cell tumors (disseminated cutaneous mast cell tumors, diffuse cutaneous mastocytosis and maculopapular cutaneous mastocytosis), histiocytic tumors (cutaneous histiocytosis [similar to the disease in dogs] and progressive histiocytosis [similar to the disease in cats]), plasma cell dyscrasia affecting the skin (cutaneous plasma cell tumors [plasmacytoma] or cutaneous involvement in multiple myeloma [similar to what rarely occurs in humans]), and lymphoid tumors (non-epitheliotropic cutaneous lymphoma). The presence of cytoplasmic granules in histology, and immunostaining for CD117, allowed us to rule out tumors of histiocytic and lymphoid origin (lymphoma and plasm cell tumors).

Diffuse cutaneous mastocytosis (DCM) is a mast cell disease described in humans (Lange et al. 2021; Rydz et al. 2024), dogs (Yang et al. 2023) and in a cat (Brown and Chalmers 1990), characterized by an extensive infiltration of MCs throughout the entire skin in the absence of macroscopic individualized cutaneous lesions. These lesions are characterized by erythroderma and pachydermia with pronounced dermographism and/or multifocal coalescing plaques, papules, vesicles, hemorrhagic bullae, and crusts (Lange et al. 2021; Rydz et al. 2024; Yang et al. 2023). Histologically, neoplastic mast cells form continuous bands from the dermal-epidermal junction to the deep dermis (Brown and Chalmers 1990; Gross et al. 2005; Kiupel 2017; Rydz et al. 2024; Yang et al. 2023). In this case, the cell distribution pattern, clinical presentation, and the infiltration of mast cells into deeper tissues (subcutaneous tissue and adjacent muscles) were the fundamental criteria for excluding the diagnosis of DCM, although both conditions present similar cell morphology.

Maculopapular cutaneous mastocytosis (MPCM) has been described in humans, especially in children under six months of age (Matito et al. 2018), cats (Chastain et al. 1988; Ngo et al. 2018), and in a dog (Davis et al. 1992). At least two forms of the disease occur in humans and animals: monomorphic and polymorphic variant. In monomorphic variant (mMPCM), which in humans occurs only in children, is characterized by larger macular, papular, plaque-type or nodular lesions brown to red heterogeneous lesions of different size, usually with a more asymmetric distribution (Matito et al. 2018). In cats, the animal species in which the disease is best described, small papules may occur that can coalesce and become covered by crusts in this form of the disease (Ngo et al. 2018). In polymorphic variant (pMPCM), which also occurs in adults in humans, is characterized by small, round, brown or red maculopapular lesions involvement of the head, particularly the lateral parts of the forehead, and the neck and extremities, palms and soles (Matito et al. 2018). In cats, the animal species in which the disease is best described, the lesions occur mainly on the front of the body front of the body (head, shoulders, ventral neck, and axillae), without any hyperpigmentation in this form of the disease (Ngo et al. 2018).

In cats, a third presentation of the MPCM has been described, characterized by more chronic and generalized lesions, mainly consisting of erythematous, crusted coalescing papules, wheals, excoriations, hyperpigmentation and lichenification; this last clinical form, the only one with clear hyperpigmentation, was typically previously described as “urticaria pigmentosa” (Ngo et al. 2018). In the histology of MPCM, all the different variants of disease revealed well-differentiated mast cells with visible granules and the dermal mast cell infiltrates are arranged in ribbons and bands, perivascular to interstitial aggregates, or coalescing aggregates forming a little dense nodule. KIT staining pattern is membranous (Matito et al. 2018; Ngo et al. 2018; Wyatt et al. 2024). These findings from the immunohistochemistry are identical to those observed in the case described here. In this case, the absence of spontaneous regression, the clinical presentation in the form of tumors (nodules, plaques, and masses), and the infiltration of mast cells into deeper tissues were the fundamental criteria for excluding the diagnosis of MPCM.

This case report describes disseminated cutaneous mast cell tumors in a South American coati, an uncommon presentation in domestic mammals (Gross et al. 2005; Kiupel 2017), that has not been previously described in this species.

## Data Availability

No datasets were generated or analysed during the current study.
